# First molecular insight into *Ovar-DRB1* exon 2 in Edilbay sheep: High heterozygosity and detection of novel variants

**DOI:** 10.14202/vetworld.2025.3959-3967

**Published:** 2025-12-18

**Authors:** Saida N. Marzanova, Davud A. Devrishov, Vladislav A. Zuchkov, Nurbiy S. Marzanov, Elizaveta A. Nikolaeva

**Affiliations:** 1 Department of Immunology and Biotechnology, Moscow State Academy of Veterinary Medicine and Biotechnology named after K. I. Skryabin, 109472, Akademika Skryabina str., 23, Moscow, Russia; 2 Center for Biotechnology and Applied Immunology, Moscow State Academy of Veterinary Medicine and Biotechnology named after K. I. Skryabin, 109472, Akademika Skryabina str., 23, Moscow, Russia; 3 Laboratory of Molecular Genetics Farm Animals, Federal Research Center for Animal Husbandry named after Academy Member L.K. Ernst, 142132, Dubrovitsy 60, Podolsk Municipal District, Moscow Region, Russia

**Keywords:** Edilbay sheep, genetic diversity, immunogenetics, Kazakh sheep breed, major histocompatibility complex, novel alleles, *Ovar-DRB1* exon 2, polymorphism, Sanger sequencing, sheep immune genetics

## Abstract

**Background and Aim:**

The *Ovar-DRB1* gene, a key component of the sheep main histocompatibility complex (MHC) class II region, plays a critical role in antigen presentation and immune responsiveness. Despite the well-documented hypervariability of exon 2 in many sheep breeds, no study has yet examined DRB1 allelic composition in Edilbay sheep, a Kazakh breed highly adapted to harsh continental steppe conditions. This study aimed to characterize the diversity of Ovar-DRB1 exon 2 alleles in Edilbay sheep and to identify novel allelic variants using Sanger sequencing.

**Materials and Methods:**

Blood samples from 50 Edilbay sheep reared at a breeding farm in Kazakhstan were subjected to DNA extraction and DRB1 exon 2 amplification using validated primers. Polymerase chain reaction products were purified and sequenced using Sanger sequencing. Allele identification was performed through pairwise sequence alignment in SnapGene and reference comparison with the Immuno Polymorphism Database of Major Histocompatibility Complex (IPD-MHC) database. Ambiguous chromatograms and overlapping nucleotide peaks were assessed for potential novel allelic patterns. Genetic diversity indices (Ho, He, Ne, and Shannon’s H’) were calculated.

**Results:**

A total of 25 known DRB1 alleles were identified in the Edilbay sheep population. Genetic diversity parameters demonstrated extremely high immunogenetic variation, with observed heterozygosity (Ho) of 0.94, expected heterozygosity (He) of 0.90, an effective number of alleles (Ne) of 16.7, and Shannon’s index (H’) of 3. Several chromatograms showed overlapping peaks or substitution patterns inconsistent with known alleles, including variations at positions 243–244 and multiple additional polymorphic sites. These patterns indicate the presence of putative novel alleles that could not be unambiguously assigned by direct Sanger sequencing. Approximately 20% of samples contained undocumented variants or low-quality chromatograms requiring further resolution.

**Conclusion:**

This study presents the first comprehensive molecular characterization of *Ovar-DRB1* exon 2 in Edilbay sheep, revealing exceptionally high genetic diversity and strong evidence for previously undescribed alleles. These findings broaden the catalog of DRB1 variants and highlight the breed’s adaptive immunogenetic potential. Further investigations using allele-specific amplification, cloning, or next-generation sequencing are recommended to precisely identify novel variants and explore associations with disease resistance and environmental adaptation.

## INTRODUCTION

The *Ovar-DRB1* gene represents one of the principal functional loci within the ovine MHC class II complex and plays a central role in shaping immune responsiveness in sheep (*Ovis aries*). It encodes the β-chain of the OLA-DR molecule, a component of the peptide-binding groove expressed on antigen-presenting cells. Through its involvement in antigen presentation to CD4^+^ T lymphocytes, the DRB1 locus directly influences the magnitude and specificity of adaptive immune responses [1]. The extensive polymorphism characteristic of MHC class II genes, including *Ovar-DRB1*, broadens the spectrum of pathogen-derived peptides that can be presented, thereby enhancing resilience against infectious pressures [2]. Several studies have linked specific DRB1 variants to favorable immune phenotypes, such as resistance to tumor development or reduced susceptibility to gastrointestinal nematode infections [3–7].

Among its exonic regions, exon 2 is the most polymorphic, encoding the hypervariable antigen-binding domain responsible for peptide recognition. More than 100 Ovar-DRB1 alleles have been documented globally, with most sheep populations typically harboring between 10 and 30 alleles. For instance, 14 distinct variants have been described in Iranian fat-tailed sheep [8]. Despite this high degree of variability, advances in molecular genotyping have facilitated the discovery of novel allelic forms. Recent investigations reported 27 previously undescribed haplotypes across six native Turkish sheep breeds [9], and a unique 2-bp deletion in DRB1 was shown to increase susceptibility to Visna-Maedi infection, suggesting strong evolutionary selection pressures on this locus [10].

The Edilbay sheep, a fat-tailed breed originating in Kazakhstan, is particularly notable for its exceptional physiological adaptation to the region’s extreme continental climate. These animals exhibit remarkable endurance, early maturity, substantial fat reserves, and the capacity to traverse long distances under harsh steppe conditions, including severe winter frosts, heavy winds, intense heat, and prolonged summer drought. Genetic studies using microsatellite markers indicate that the Edilbay-1 line has the highest allelic richness among Kazakh sheep populations, averaging 8.3 alleles per locus. Furthermore, analyses based on protein loci, microsatellite markers, and erythrocyte antigens consistently position Edilbay sheep as a distinct genetic cluster, separate from North Caucasian indigenous breeds [11, 12].

Despite extensive characterization of the *Ovar-DRB1* exon 2 polymorphism in many sheep breeds across Asia, Europe, and the Middle East, no study has reported the allelic composition of this locus in Edilbay sheep, a genetically distinct and ecologically resilient Kazakh breed. Current literature demonstrates high DRB1 diversity in Turkish, Iranian, and Sudanese breeds, including the discovery of numerous novel haplotypes; however, the immunogenetic structure of Edilbay sheep remains entirely undocumented, despite their unique adaptation to the harsh continental steppe environment. Microsatellite-based assessments indicate that Edilbay sheep exhibit exceptionally high allelic richness and form a distinct genetic cluster, strongly suggesting the potential presence of rare or breed-specific MHC variants. Yet, no sequence-based analyses of DRB1 exon 2 have been conducted to confirm this, limiting our understanding of how immunogenetic diversity contributes to the breed’s resilience to pathogens, extreme climate, and ecological stressors. Moreover, the absence of DRB1 data for Edilbay sheep hinders comparative immunogenetic analyses and restricts the development of marker-assisted breeding strategies tailored to this economically valuable population. This gap underscores the urgent need to molecularly characterize DRB1 exon 2 in Edilbay sheep to identify both known and potentially novel allelic variants.

The present study was designed to characterize, for the first time, the allelic diversity of *Ovar-DRB1* exon 2 in Edilbay sheep from Kazakhstan using direct Sanger sequencing. Specifically, the study aimed to (i) identify known DRB1 alleles circulating within the Edilbay population through pairwise sequence alignment with the Immuno Polymorphism Database of Major Histocompatibility Complex (IPD-MHC) reference database; (ii) detect potential novel or undocumented polymorphic sites through the evaluation of ambiguous chromatogram peak patterns; and (iii) assess key genetic diversity indices, including observed and expected heterozygosity, effective allele number, and Shannon’s information index, to quantify the immunogenetic variability of the population. By integrating sequence-based genotyping with diversity metrics, this research sought to generate the first molecular insight into DRB1 exon 2 variation in Edilbay sheep, provide evidence for putative new allelic variants, and establish a foundational dataset for future investigations into disease resistance markers, adaptive evolution, and selective breeding strategies in this historically important Kazakh breed.

Currently, no publications have reported the direct typing of DRB1 exon 2 in Edilbay sheep. Nevertheless, this breed is a valuable subject of investigation because of its exceptional adaptation to the harsh nomadic conditions of the Kazakh steppe. The breed’s high genetic variability and active selection make DRB1 allele screening a promising approach. This study aimed to characterize the allelic composition of the *Ovar-DRB1* exon 2 in Edilbay sheep from the Kuanysh peasant farm in Kazakhstan and to identify previously undescribed DRB1 alleles in the gene pool. Analysis of novel and rare DRB1 alleles is important for evaluating the herd’s immune potential and selecting breeding animals with favorable immunogenetic profiles.

## MATERIALS AND METHODS

### Ethical approval

All procedures involving animals in this study were conducted in accordance with established veterinary welfare regulations and institutional guidelines for the ethical use of farm animals in research. Blood sampling was performed as part of routine herd health monitoring at the Kuanysh peasant farm (Akzhaik district, Republic of Kazakhstan), and no experimental interventions, treatments, or procedures outside normal veterinary practice were applied.

The study did not involve any invasive experimental manipulation, clinical trial, animal handling beyond standard restraint, or procedures likely to cause pain, distress, or long-term impact. Jugular venipuncture was carried out by licensed veterinary personnel using aseptic techniques to minimize discomfort. No sedation or analgesia was required, and no animals were harmed or removed from production as a result of this research.

According to local regulations governing observational genetic studies based on biological samples collected during routine management, dedicated institutional ethics committee approval is not mandatory. Nevertheless, the research team followed all relevant guidelines for humane treatment, including the principles of the European Convention for the Protection of Vertebrate Animals Used for Experimental and Other Scientific Purposes (ETS No. 123) and the Guidelines for the Care and Use of Agricultural Animals in Research and Teaching.

Permission to use the biological material and associated data was obtained from the farm management, and informed consent for sampling was provided by the animal owners. All laboratory procedures were performed using anonymized samples and under biosafety conditions appropriate for molecular genetic studies.

### Study period and location

The study was conducted from April 8 to July 18, 2025, at Moscow State Academy of Veterinary Medicine and Biotechnology named after K. I. Skryabin, Moscow, Russia.

### Animals

A total of 50 Edilbay sheep, each 2.5 years old, were selected from the Kuanysh peasant farm located in the Akzhaik district of the Republic of Kazakhstan. The farm maintains a breeding population of more than 2,500 Edilbay sheep. In this region, adult Edilbay ewes at 2.5 years of age typically weigh between 65 and 80 kg.

### Collection of biological material

Whole blood samples were collected by jugular venipuncture and transferred into 6-ml tubes containing 0.05% ethylenediaminetetraacetic acid (EDTA). Samples were immediately frozen at −20°C until DNA extraction.

Genomic DNA was isolated using the EX-516 sorbent-based commercial kit (“SINTOL”, Moscow, Russia), following the manufacturer’s protocol. DNA was eluted in 100 μL of buffer and stored at −20°C. DNA concentration and purity were assessed using a NanoDrop OneC spectrophotometer (Thermo Scientific, USA). Samples with A260/280 ratios between 1.8–2.0 and A260/230 ratios above 2.0 were accepted for analysis; samples outside these ranges were either discarded or re-extracted.

**Polymerase chain reaction** (PCR) Amplification and Sequencing of DRB1 Exon 2

Amplification of *Ovar-DRB1* exon 2 was performed using previously validated primers [13] covering the complete variable region:

- Forward primer (330F): 5′-ATTAGCCTCYCCCCAGGAGKC-3′
- Reverse primer (DRB1_E2_rev): 5′-GCTCACCTCGCCGCT-3′

Each primer was used at a final concentration of 500 nM.

PCR cycling conditions consisted of:

- Denaturation: 95°C for 30 s
- Annealing: 58°C for 30 s
- Extension: 72°C for 30 s for a total of 30 cycles.

PCR products were visualized on 1.5% agarose gels stained with SYBR™ Gold (Thermo Fisher Scientific, USA). Target amplicons were purified using the CleanUp mini kit (“Eurogen”, Russia). Purified PCR fragments were sequenced by “Eurogen”, Russia, using Sanger sequencing on a 3500xL Genetic Analyzer (Applied Biosystems, USA), with expected read lengths of 700–800 nucleotides.

### Sequencing data analysis and allele typing

Sequence analysis and allele identification were conducted using SnapGene 2.8.3 (USA) with pairwise alignment performed via the Needleman–Wunsch algorithm. Allele interpretation was based on reference sequences from the IPD-MHC OLA database (https://www.ebi.ac.uk/ipd/mhc/group/OLA/ version as of June 1, 2025) [14].

Samples showing amplification of two alleles exhibited chromatograms with overlapping peaks. All known DRB1 exon 2 reference sequences were aligned with each obtained chromatogram. Alleles with mismatched nucleotides that showed clear chromatogram peaks were excluded. For ambiguous chromatograms, possible allele pair combinations were evaluated to determine the most consistent match.

- 40 samples were assigned unambiguous allele combinations.
- 10 samples could not be conclusively typed due to low-quality chromatograms or atypical substitution patterns.

### Statistical analysis

Allele frequencies and graphical outputs were generated using Microsoft Excel 2023 (Microsoft Corp., Washington, USA). Calculations were performed as follows:

### Allele Frequency


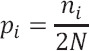


where:

- *n_i_*= number of copies of allele *i*
- *N*=number of individuals (each carrying two alleles)

Observed Heterozygosity (Ho)


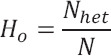


where *N_het_*= number of heterozygous individuals.

Expected Heterozygosity (He)


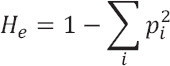


Effective Number of Alleles (Ne)


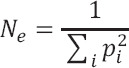


Shannon’s Information Index (H′)


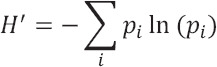


## RESULTS

### PCR amplification and sequencing quality

Amplification of the DRB1 exon 2 region was successful for all examined animals. PCR efficiency was confirmed through horizontal agarose gel electrophoresis, after which target DNA bands were excised, purified, and subjected to Sanger sequencing. Sequencing produced high-quality, unambiguous chromatograms for 80% of samples, which were included in allele identification. The remaining 20% of samples showed undocumented nucleotide patterns or low-quality chromatograms and were therefore excluded from downstream allele typing.

### Genetic diversity and allelic composition

Exceptionally high genetic diversity was observed at the Ovar-DRB1 locus in Edilbay sheep. The observed heterozygosity (Ho) was 0.94, while the expected heterozygosity (He) reached 0.90. A total of 25 distinct alleles were identified, and the effective number of alleles (Ne) was calculated as 16.7. This elevated Ne value suggests a relatively even distribution of allele frequencies with no strong dominance of specific variants, consistent with the known hyperpolymorphic nature of DRB1.

Although several samples were unsuitable for interpretation, likely due to contamination or suboptimal PCR conditions that produced mixed chromatogram signals, many alleles matched reference sequences in the IPD-MHC database.

**Figure 1:**
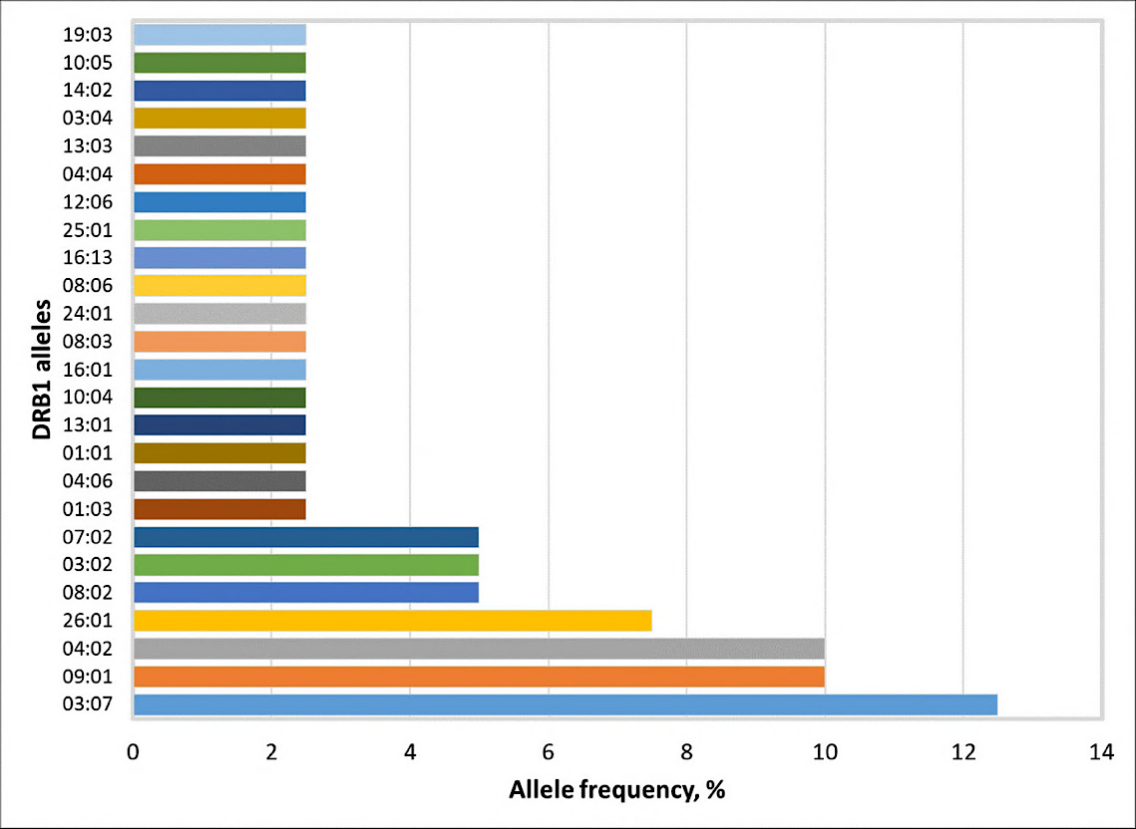
Frequency distribution of the detected DRB1 alleles.

Across the population, the following DRB1 exon 2 alleles were detected: 08:02, 01:03, 03:07, 04:06, 01:01, 09:01, 04:02, 13:01, 10:04, 26:01, 03:02, 16:01, 07:02, 08:03, 24:01, 08:06, 16:13, 25:01, 12:06, 04:04, 13:03, 03:04, 14:02, 10:05, and 19:03. The distribution of allele frequencies is presented in Figure 1 and Table 1.

**Table 1:** Allele frequencies and immunopolymorphism database of major histocompatibility complex accessions of the DRB1 variants.

Allele	Frequency, %	IPD-MHC accession	Allele	Frequency, %	IPD-MHC accession
03:07	12.5	OLA02472	08:03	2.5	OLA02465
09:01	10	OLA02428	24:01	2.5	OLA04078
04:02	10	OLA02459	08:06	2.5	OLA08690
26:01	7.5	OLA08709	16:13	2.5	OLA08873
08:02	5	OLA02451	25:01	2.5	OLA08670
03:02	5	OLA02424	12:06	2.5	OLA08878
07:02	5	OLA02457	04:04	2.5	OLA08684
01:03	2.5	OLA02464	13:03	2.5	OLA08672
04:06	2.5	OLA08711	03:04	2.5	OLA02454
01:01	2.5	OLA02426	14:02	2.5	OLA02782
13:01	2.5	OLA02452	10:05	2.5	OLA08675
10:04	2.5	OLA08669	19:03	2.5	OLA02475
16:01	2.5	OLA02456			

### Detection of atypical or novel nucleotide patterns

**Figure 2:**
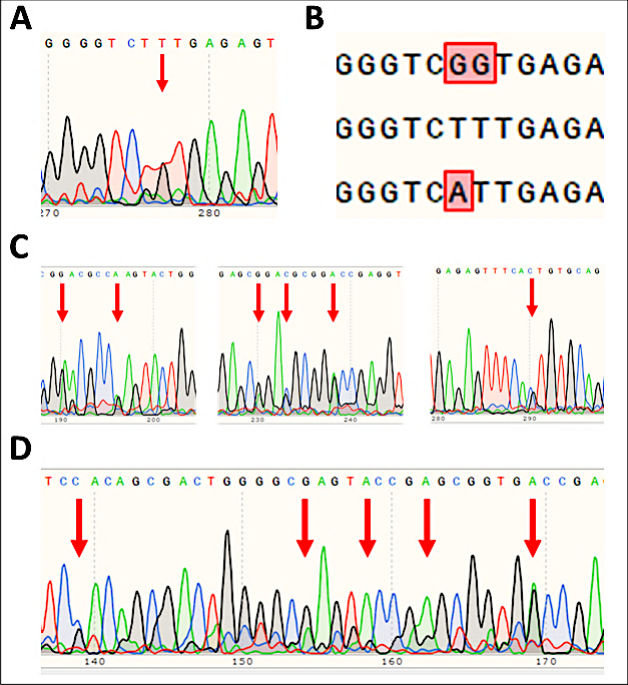
Atypical nucleotide substitution patterns in the DRB1 exon 2 samples. (A) Chromatogram showing substitutions at positions 243 and 244. (B) Possible nucleotide substitution variants among known alleles. (C) Nucleotide substitution pattern in sample 11. (D) Nucleotide substitution pattern in sample 21.

Several chromatograms displayed nucleotide substitution patterns that did not match any known allele combinations recorded in the IPD-MHC database. The most frequent anomaly involved a dual peak (T/G) at nucleotide position 244 of exon 2 (codon 115), while position 243 consistently showed a single T peak (Figure 2A). This pattern does not correspond to any pair of known alleles, as allele combinations with G at position 244 typically also exhibit G at position 243 (Figure 2B).

These sequence patterns (Figure 2D) also support the likelihood of novel Ovar-DRB1 exon 2 variants.

Additional atypical substitutions were recorded at positions:

- 156 A/G (codon 86)
- 162 A/G (codon 88)
- 196 A/G (codon 99)
- 199 G/C (codon 100)
- 204 A/G (codon 102)
- 256 G/C (codon 119)

These patterns suggest the presence of one or more previously undescribed allelic variants within the Edilbay sheep population (Figure 2C).

A further group of ambiguous chromatograms included substitutions at:

- 108 G/C (codon 70)
- 123 A/G (codon 75)
- 127 A (codon 76)
- 131 A (codon 77)
- 138 A/G (codon 80)

### Need for further molecular resolution

Given the limitations of direct Sanger sequencing in resolving heterozygous positions in highly polymorphic loci, these atypical substitution profiles require follow-up investigation using higher-resolution approaches. Techniques such as allele-specific amplification, PCR fragment cloning, or next-generation sequencing (NGS) will be essential for precisely characterizing these putative novel DRB1 variants.

## DISCUSSION

### Allelic diversity and immunogenetic structure of the population

The identification of 25 allelic variants of the *Ovar-DRB1* gene in Edilbay sheep provides substantial insight into the breed’s immunogenetic composition. High allelic diversity at the DRB1 locus is a hallmark of MHC class II genes across sheep populations, reflecting their essential role in pathogen recognition and adaptive immunity [9]. Polymorphisms in exon 2, in particular, directly affect the structure of the antigen-binding groove, enabling individuals to recognize a broad spectrum of pathogenic peptides [3, 8, 15]. This mechanism underlies population-level resistance to diverse infectious agents and enhances long-term resilience.

The exceptionally high heterozygosity observed in this study indicates strong immunogenetic variability in Edilbay sheep. Such diversity likely results from adaptation to a wide range of environmental pathogens, supported by the breed’s historically broad genetic base and possible gene flow among subpopulations. Elevated DRB1 variability is highly advantageous for breeding and conservation programs, as it enhances the overall antigen recognition repertoire and contributes to improved resistance against endemic diseases.

### Comparison with other regional and international breeds

Several alleles detected in the Edilbay population, such as 01:01, 03:02, and 04:02, are known to be widespread in other sheep breeds and have been historically associated with advantageous immune traits, including resistance to infectious diseases [2, 10, 16]. Similar associations have been reported across multiple species for allele families DRB*01, DRB*03, and DRB1*04, underscoring the functional relevance of these variants [17, 18].

The total number of alleles detected (n = 25) aligns closely with reports from other geographically and ecologically challenging breeds, such as local Turkish sheep, which exhibit similar levels of DRB1 diversity [9, 19]. Additionally, the Edilbay sheep demonstrated higher observed heterozygosity (Ho = 0.94) and expected heterozygosity (He = 0.90) than several regional breeds, such as the Iranian Lori-Bakhtiar sheep (Ho = 0.82), indicating exceptionally high immunogenetic variability.

The effective allele number (Ne = 16.7) further reflects a balanced allele frequency distribution consistent with strong balancing selection, likely driven by pathogen-mediated pressures. Although Sudanese Desert Sheep show higher overall allelic counts (46 alleles, including four novel variants) [20], the Edilbay breed’s diversity remains noteworthy given its distinct ecological niche and evolutionary history. The identification of putative novel allele patterns in approximately 10% of samples strengthens the evidence that Edilbay sheep may harbor unique immunogenetic traits relevant to adaptation and disease resistance.

### Evidence for novel and previously undescribed allelic variants

Multiple chromatograms displayed nucleotide substitution patterns inconsistent with any known DRB1 sequences, suggesting the presence of previously undescribed allelic variants within the Edilbay population. These findings were observed across several samples, indicating that such variants are unlikely to be artifacts and may reflect true, population-specific polymorphisms.

Direct Sanger sequencing, while widely used, is limited in resolving overlapping nucleotide peaks in heterozygous MHC loci, which frequently exhibit high variability. Mixed chromatogram peaks arise when two alleles are amplified simultaneously, complicating allele assignment [21, 22]. This challenge is well-documented in studies of highly polymorphic genes such as *Ovar DRB1*.

Given these constraints, further resolution of novel alleles will require more advanced methods, including:

- Cloning of PCR fragments followed by sequencing of individually isolated alleles [23],
- Allele-specific primers to selectively amplify candidate variants [24],
- Targeted NGS for high-depth, high-accuracy allele reconstruction [25].

These approaches will enable the accurate determination of new allele sequences, improve the characterization of MHC diversity, and facilitate the deposition of validated sequences into international databases.

### Implications for Breed Adaptation and Evolutionary Immunogenetics

The presence of putative novel DRB1 variants highlights the Edilbay breed as a valuable genetic resource for understanding adaptive evolution in harsh continental climates. Each novel allele may encode unique antigen-binding properties that enhance the recognition of local pathogens [8, 26]. Similar patterns have been observed in other populations that evolved under strong environmental pressures, where up to 46% of MHC alleles within a single population were newly identified [20, 27].

The distinct climatic conditions of Kazakhstan, including extreme temperature fluctuations, limited forage availability, and diverse pathogen exposures, likely shaped the immunogenetic architecture of Edilbay sheep through long-term natural and artificial selection. Consequently, the allelic composition of DRB1 in this breed holds significant implications for marker-assisted selection, disease-resistance breeding, and conservation of adaptive traits.

### Overall Significance

Collectively, the extensive DRB1 diversity and the evidence of previously undescribed allelic variants underscore the exceptional adaptive capacity of Edilbay sheep. These findings provide a strong foundation for future research into immunogenetic markers associated with resilience to infectious diseases and environmental stressors. Moreover, the identification of novel DRB1 variants reinforces the importance of conserving genetically distinct regional breeds and integrating immunogenetic data into breeding programs.

## CONCLUSION

This study provides the first molecular characterization of *Ovar-DRB1* exon 2 allelic diversity in Edilbay sheep and reveals a highly polymorphic immunogenetic profile for this Kazakh breed. A total of 25 distinct DRB1 alleles were identified, accompanied by exceptionally high observed heterozygosity (Ho = 0.94), expected heterozygosity (He = 0.90), and an effective allele number (Ne = 16.7). These metrics confirm that Edilbay sheep possess one of the most diverse DRB1 repertoires among regional breeds, reflecting strong pathogen-mediated selection and long-term adaptation to Kazakhstan’s harsh continental environment. Importantly, several chromatogram patterns did not match any known IPD-MHC alleles, indicating the presence of novel, previously undescribed DRB1 variants within this population.

The findings have practical significance for improving breeding programs, as high MHC variability enhances the capacity for broad antigen recognition and may be leveraged in marker-assisted selection to strengthen disease resistance and resilience in Edilbay sheep. The study’s strengths include the application of sequence-based genotyping, evaluation of naturally reared animals, and integration of multiple diversity indices, all of which provide a comprehensive assessment of DRB1 variation. However, direct Sanger sequencing imposes limitations in resolving heterozygous or novel alleles due to overlapping chromatogram peaks, which prevent full allele identification in approximately 20% of samples. Advanced methods such as cloning, allele-specific amplification, or NGS will be needed to accurately characterize the putative novel variants detected in this study.

Future research should focus on validating the newly observed nucleotide substitution patterns, elucidating their functional relevance, and exploring associations between specific DRB1 alleles and phenotypic traits such as disease resistance, parasite tolerance, reproductive performance, and environmental adaptation. Such work will deepen the understanding of the immunogenetic architecture of Edilbay sheep and support the development of resilient breeding lines optimized for challenging steppe environments.

Overall, this study highlights the exceptional immunogenetic diversity of Edilbay sheep and their potential as a valuable source of novel MHC variation. These findings provide meaningful new insights into livestock immunogenomics and underscore the importance of conserving genetically unique regional breeds that harbor rare, potentially adaptive immune alleles.

## DATA AVAILABILITY

All the generated data are included in the manuscript.

## AUTHORS’ CONTRIBUTIONS

DAD, NSM, and SNM: Conceptualization, project administration, data analysis, and drafted and edited the manuscript. SNM, VAZ, and EAN: Data acquisition and statistical analysis. VAZ, NSM, and SNM: Data analysis and drafted the manuscript. All authors have read and approved the final version of the manuscript.

## ACKNOWLEDGMENTS

This study was supported by the Russian Science Foundation (grant no. 24-26-00197, https://rscf.ru/ project/24-26-00197/). The authors are thankful to Dr. Kairly G. Yessengaliyev and Dr. Ainur M. for their assistance in blood sampling. Davletova (West Kazakhstan Innovation and Technology University, Uralsk, Kazakhstan), Ersain S. Nysanov (West Kazakhstan Scientific Veterinary Station, branch of Kazakh Scientific Research Veterinary Institute, LLP, Uralsk, Kazakhstan).

## COMPETING INTERESTS

The authors declare that they have no competing interests.

## PUBLISHER’S NOTE

Veterinary World remains neutral with regard to jurisdictional claims in the published institutional affiliations.
